# Microscopic description of insulator-metal transition in high-pressure oxygen

**DOI:** 10.1038/s41598-017-02730-z

**Published:** 2017-06-01

**Authors:** Luis Craco, Mukul S. Laad, Stefano Leoni

**Affiliations:** 1 Instituto de Física, Universidade Federal de Mato Grosso, 78060-900 Cuiabá, MT Brazil; 2 The Institute of Mathematical Sciences, C.I.T. Campus, Chennai, 600 113 India; 3 School of Chemistry, Cardiff University, Cardiff, CF10 3AT UK

## Abstract

Unusual metallic states involving breakdown of the standard Fermi-liquid picture of long-lived quasiparticles in well-defined band states emerge at low temperatures near correlation-driven Mott transitions. Prominent examples are ill-understood metallic states in *d*- and *f*-band compounds near Mott-like transitions. Finding of superconductivity in solid O_2_ on the border of an insulator-metal transition at high pressures close to 96 GPa is thus truly remarkable. Neither the insulator-metal transition nor superconductivity are understood satisfactorily. Here, we undertake a first step in this direction by focussing on the pressure-driven insulator-metal transition using a combination of first-principles density-functional and many-body calculations. We report a striking result: the finding of an orbital-selective Mott transition in a pure *p*-band elemental system. We apply our theory to understand extant structural and transport data across the transition, and make a specific two-fluid prediction that is open to future test. Based thereupon, we propose a novel scenario where soft multiband modes built from microscopically coexisting itinerant and localized electronic states are natural candidates for the pairing glue in pressurized O_2_.

## Introduction

The unique properties of high-pressure induced solid phases of molecular gases continue to evince keen and enduring interest in condensed matter physics. Beginning with early ideas of Mott^1^ and extending up to modern times^2^, ideas of pressure-induced electronic, magnetic and structural transitions and possible superconductivity in such systems even provided early ground for strongly correlated systems, are currently a frontline research topic in condensed matter. Particularly interesting examples of intriguing physics in solidized molecular phases of gases are dense hydrogen^3^ and solid oxygen^4, 5^, as well as the most recent report of very high-*T*_*c*_ superconductivity in solid H_2_S under very high pressure^6^. H_2_ is predicted to metallize under high pressure, while solid O_2_ even shows a superconducting phase (*T*_*c*_ = 0.6 K) at the border of a pressure-driven transition from a non-magnetic insulator to paramagnetic metal, joining the long list of materials exhibiting superconductivity proximate to metal-insulator transitions.

**Figure 1:**
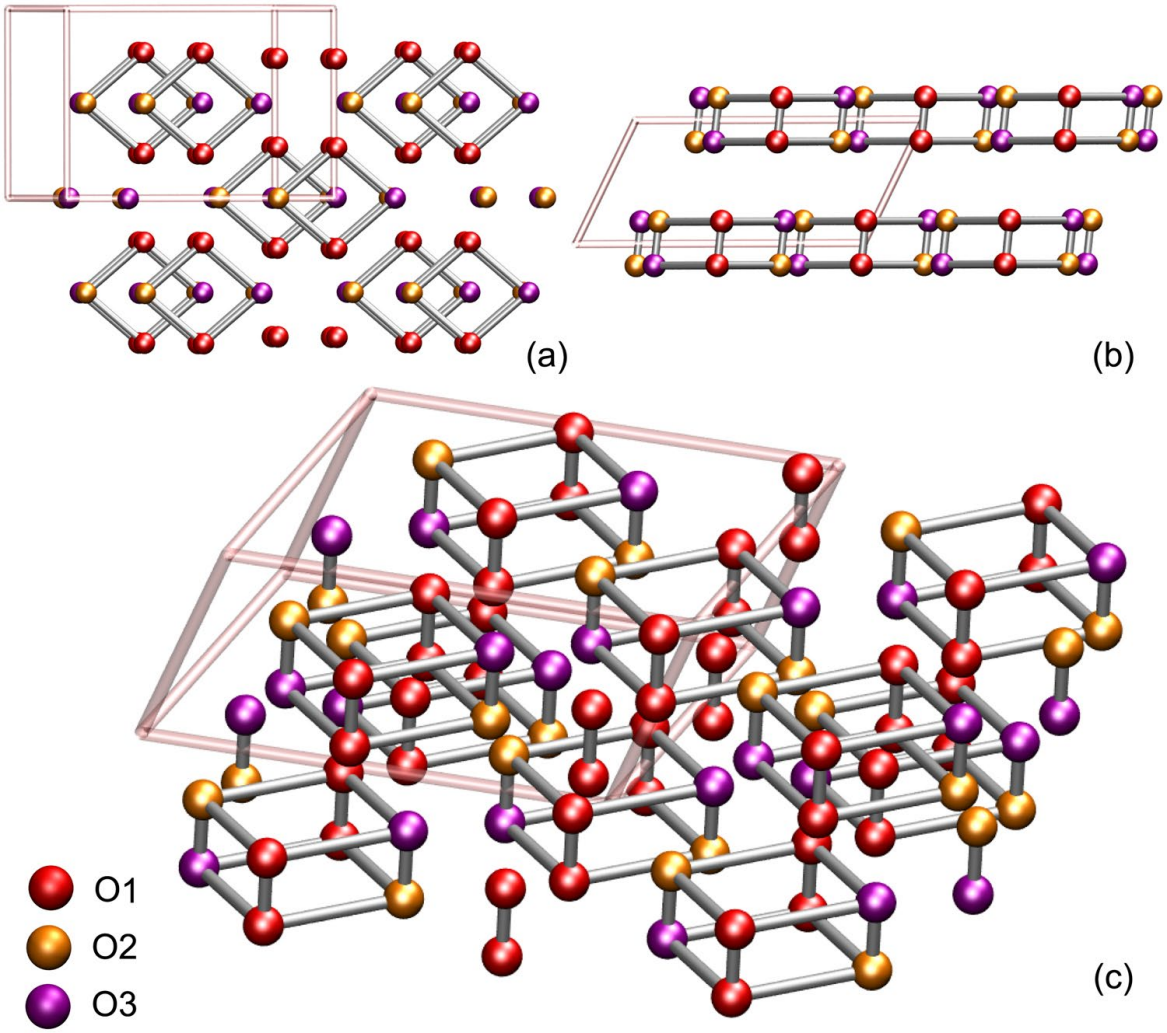
Crystal structure of the *ε*-phase of solid oxygen. The structure as viewed perpendicular to the **a**–**b** (**a**) and **a**–**c** (**b**) planes. The O_8_ clusters in the monoclinic unit cell (rose lines) are shown in (**c**). O*x*(*x* = 1, 2, 3) label the three inequivalent oxygen atoms.

Pressurized molecular oxygen forms various low-temperature solid phases under pressure, labelled *α*, *δ*, *ε* and *ζ* phases^7^. At lower pressure, the antiferromagnetically ordered *α* phase transforms into another antiferromagnetically ordered *δ* phase at 5.4 GPa, followed by a non-magnetic *ε* phase at 8 GPa. Higher pressure, $P\simeq 96$ GPa, metallizes the system^8^, followed by emergence of superconductivity below ${T}_{c}\simeq 0.6$ K^9^. This astounding behavior in a molecular system, reminiscent of strongly correlated, doped Mott insulators in *d*-band oxides like cuprates, presents a significant challenge for theory. The high-*P ε*–*ζ* phase transition is also accompanied by significant volume reduction^10^, with a contraction of about 10% of the lattice parameter along the *b* direction. The *ε* phase retains the layered nature of the lower pressure phases^4^, and the monoclinic (*C*2/*m*) structure^10, 11^ as shown in Fig. 1.

That the driving force for the *α*–*β* transition at moderate *T* is dominantly magnetic has been established in a series of careful studies^12–14^. Indeed, early work of da Silva and Falicov^15^ already explained the measured heat of formation at the *α*–*β* transition in terms of the entropy difference computed from cluster analysis of a multi-orbital Hubbard model (or an equivalent *S* = 1 Heisenberg-like model in *d* = 2 dimensions). Observation of very different magnetic orders in the *α*, *β* phases, correlation between magnetic and structural changes along with ferromagnetic coupling between the off-plane near neighbors in the *δ* phase are reminiscent of those found in classic multi-band systems like V_2_O_3_^16^, taken together with the above, favor a multi-orbital description. Additional evidence for multi-orbital effects is provided by the anisotropic and partially discontinuous pressure-induced changes in the lattice parameters in the different phases^11, 17^. In such a scenario, increasing pressure is expected, in the simplest approximation, to decrease lattice spacings and increase the carrier itinerance. The result would then be to suppress antiferromagnetic order along with insulating behavior, and to induce metalization. In solid O_2_, antiferromagnetic order is destroyed well before metalization occurs^18^, and so, within the *p* ^4^ configuration of oxygen, the insulator-metal transition across the *ε*–*ζ* transition must be regarded as a Mott metal-insulator transition. This suggests that on the one extreme, a Heisenberg model description is only valid in the insulating *α*, *β*, *δ* phases, and that a more general multi-orbital Hubbard model must be used, at least for the *ε* phase. At the other extreme, one-electron band structure calculations for the antiferromagnetically ordered phases do provide qualitatively correct ground states^19^. In addition, electronic structure calculation based on generalized gradient approximation (GGA) shows that the nonmagnetic insulating state is energetically favored at pressures corresponding to the *ε*-phase^20, 21^. However, by construction, *ab initio* density-funtional calculations have intrinsic difficulties in describing non-magnetic insulating phases, and in particular the *ε* phase^22, 23^, for reasons described in detail in ref. 24. The observation of superconductivity at the border of this (Mott) insulator-to-metal transition thus suggests that dualistic behavior of correlated carriers (see our discussion below) near the insulator-metal transition is very likely implicated in the pairing glue. Thus, a search for the microscopic origin of the pair glue must involve understanding of the insulator-metal transition around 96 GPa.

Before presenting our local-density-approximation plus dynamical-mean-field (LDA + DMFT) results, we point out essential differences between band and Mott insulators. In conventional semiconductors (or band insulators) all bands below the Fermi energy are filled and, therefore, inert. Removing an electron leads to an empty state which can be thought of as a hole moving freely through the solid. The same is true for an added electron, which occupies the first empty band. In a multi-orbital Mott-Hubbard insulator, the insulating state arises because electron hopping from one site to another is inhibited by intra- and inter-orbital Coulomb repulsions. In these systems, when the band filling is slightly reduced from its commensurate value, a small number of unoccupied states are created; similarly adding electrons creates locally doubly occupied electronic states. The crucial difference in this case is that since the doped carriers can have either spin (↑, ↓) with equal probability, doping a Mott insulator, *e*.*g*., by holes, creates two available states at the Fermi energy. This is at the heart of spectral weight transfer, a phenomenon ubiquitous to Mott, as opposed to band, insulators. In both cases, electron hopping might still be prevented by inter-orbital Coulomb interactions in a multiband system. The resulting metallic state upon doping can vary from a Fermi liquid at weak coupling to an exotic orbital-selective, non-Fermi liquid metal for stronger electron-electron interactions, as doping and temperature^25^ are varied. This fundamental difference between band and multi-orbital Mott-Hubbard insulators is of basic and practical interest. Below we show that sizable multiband electronic interactions are the clue to the insulating state of the *ε*-phase of solid oxygen and its evolution to a non-Fermi liquid metallic state at high pressures.

Possibility of Mott-Hubbard physics in purely *p* ^26–29^ or *s* ^30^ band systems is very intriguing, since the naive expectation dictates that the itinerance (kinetic energy of *p*, *s*-carriers) is appreciable compared to the electron-electron interactions, as distinct from *d*-band systems, where the *d* electrons reside in much narrower bands (hence the effective *U*/*W* is sizable; *U* and *W* are, respectively, the on-site Coulomb repulsion and the bare one-particle band width)^31^. Thus, understanding Mottness (or the proximity to a Mott-Hubbard insulating state) in materials with active *p* or *s* bands is undoubtedly an issue of great contemporary interest^32^. In light of the discussion above, we study how an orbital-selective interplay between appreciable *p*-band itinerance and sizable, on-site Coulomb repulsion, *U*, plays a central role in this unique Mott transition in solid O_2_.

## Results

### Electronic Structure

**Figure 2:**
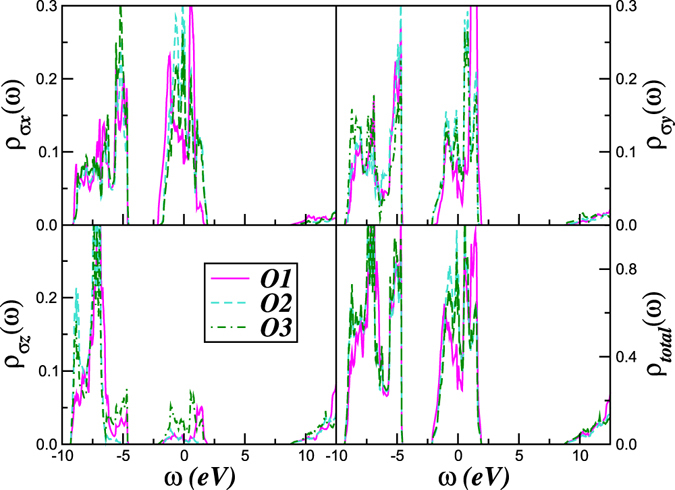
Orbital resolved and total LDA density-of-states (DOS) for the three inequivalent oxygen atoms in the *ε*-phase. Notice that all bands as well span over the Fermi level. Also relevante is the evolution of the electronic DOS at different polarizations.

To quantify the correlated electronic structure of solid O_2_, we start with the *C*2/*m* structure (Fig. 1) with lattice parameters derived in ref. 11. Here, local-density approximation (LDA) calculations for the real crystal structure of the *ε*-phase were performed using the linear muffin-tin orbitals (LMTO)^33–35^ scheme in the atomic sphere approximation^36^. The corresponding LDA density-of-states of the three (symmetry) inequivalent atoms^4, 11^ is shown in Fig. 2. Strong intramolecular overlap leads to propensity to localization of the *p*_*z*_, i.e., the *σ*-orbital^19, 37^ in the energy level diagram of O_2_. However, due to inter-molecular orbital overlap in the monoclinic structure, the *p*_*z*_ states acquire some itinerance, explaining the small amount of *p*_*z*_ states found at the Fermi energy. As seen in Fig. 2, all *π*-bands cross the Fermi energy, providing a metallic state within LDA.

Within LDA, the one-electron part of the many-body Hamiltonian for solid oxygen is now ${H}_{0}={\sum }_{{\bf{k}},a,\sigma }\,{\varepsilon }_{a}({\bf{k}}){c}_{{\bf{k}},a,\sigma }^{\dagger }{c}_{{\bf{k}},a,\sigma }+{\sum }_{i,a,\sigma }\,{{\rm{\Delta }}}_{a}{n}_{i\sigma }^{a}$, where *a* = *x*, *y*, *z* label the three diagonalized *p* orbitals and the Δ_*a*_ are on-site orbital energies in the real structure of solid O_2_. In light of antiferromagnetic insulator^15^ phases and the non-magnetic Mott transition, local multi-orbtial interactions are mandatory to understand O_2_. These constitute the interaction terms ${H}_{int}=U{\sum }_{i,a}\,{n}_{i\uparrow }^{a}{n}_{i\downarrow }^{a}+U^{\prime} {\sum }_{i,a\ne b}\,{n}_{i}^{a}{n}_{i}^{b}-{J}_{H}{\sum }_{i,a\ne b}\,{S}_{ia}\cdot {S}_{ib}$. Here, $U(U^{\prime} \equiv U-2{J}_{H})$ is the intra- (inter-) orbital Coulomb repulsion and *J*_*H*_ is the Hund’s rule term. Following da Silva and Falicov^15^, we use *U* = 11.6 eV and *J*_*H*_ = 0.45 eV, along with the LDA bands of the three inequivalent oxygen atoms described above. In this work, the correlated multi-orbital problem of solid O_2_ encoded in *H* = *H*_0_ + *H*_*int*_ is treated within the state-of-the-art local-density-approximation plus dynamical-mean-field-theory (LDA + DMFT) scheme^31^. The DMFT self-energy, ${{\rm{\Sigma }}}_{a}(\omega )$, requires a solution of the multi-orbital quantum impurity problem self-consistently embedded in an effective medium^31^. We use the multi-orbital iterated-perturbation-theory (MO-IPT) as an impurity solver for DMFT^38^: This analytic solver has a proven record of successes in describing finite temperature Mott transitions^39^ as well as unconventional behavior in correlated *p*-band systems^40–42^.

With orbital orientation-induced anisotropic LDA one-particle energies and hoppings, multi-orbital correlations renormalize various *p*-bands in different ways. Generically, one expects partial (Mott) localization of a subset of bands, leading to orbitally selective Mott transitions, and bad metallic states^31, 39, 43^. Within LDA + DMFT, this orbital-selective mechanism involves two renormalizations: static (multi-orbital Hartree) renormalization shifts the *p*-bands relative to each other by amounts depending upon their *bare* on-site orbital energies (Δ_*a*_) and occupations (*n* ^*a*^). In addition, dynamical effects of *U*, $U^{\prime} \equiv U-2{J}_{H}$ drive large spectral weight transfer over wide energy scales^39, 43^. The large, anisotropic changes in dynamical spectral weight transfer in response to small changes in bare one-particle (LDA) parameters (for example, crystal-field splittings under pressure)^39^ are known to drive the orbital-selective Mott transition in real multi-orbital systems. As we show below, precisely such an orbital-selective Mott transition, accompanied by an incoherent metallic phase in solid O_2_, occurs at very high pressures.

**Figure 3:**
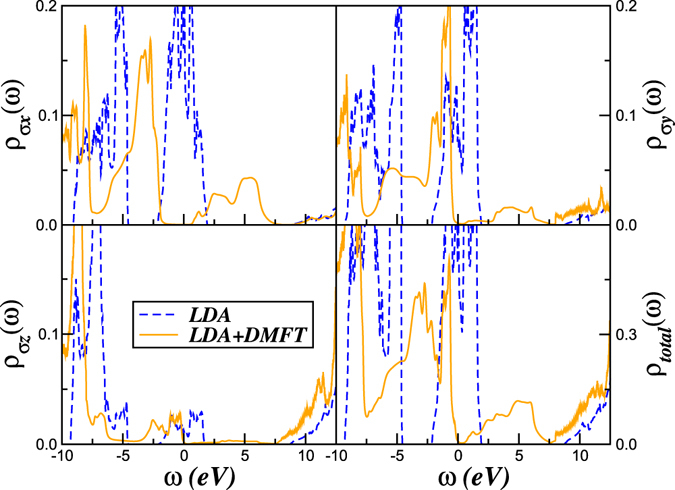
Comparison between the LDA and LDA + DMFT orbital-resolved and total density-of-states in the *ε*-phase of solid O_2_. LDA + DMFT results for the Mott insulating *ε* phase of oxygen were obtained using the intra- (inter-) orbital Coulomb repulsion, *U* = 11.6 eV (*U*′ = 10.7 eV) and the Hund’s rule interaction *J*_*H*_ = 0.45 eV for the total band filling, *n* = 4. Large-scale transfer of spectral weight from low energy to high energies is visible in the correlated spectral functions of the *p*_*x*_ and *p*_*y*_ bands. Also clear is the destruction of the low-energy peak of the *p*_*z*_ in LDA.

Using *U*, *U*′, *J*_*H*_ as obtained in ref. 15, we find that the *ε* phase is a Mott insulator, as shown in Fig. 3. The size of the charge gap is orbital dependent, and is larger for the *p*_*x*_ band compared to the other two. Large spectral weight transfer, characteristic of dynamical local correlations, is explicitly manifested in the qualitative difference between LDA and LDA + DMFT spectra. At first sight, derivation of a Mott insulator with *U* < *W* in solid O_2_ seems a bit puzzling. The reason, however, is that, in this multi-orbital system, both *U*, *U*′ are appreciable, and the combined effect of both acting in tandem is to (*i*) reduce the band-width of each band (this can arise solely from *U*, even for the artificial case of *U*′ = 0), and (*ii*) the dominant effect of *U*′ on a reduced bandwidth is to split the bands via the Mott mechanism. In the actual multi-orbital problem, both effects are simultaneously operative, and reinforce each other.

From our results in Fig. 3, we compute the renormalized orbital splittings (*δ*_*a*_) and occupations (*n* ^*a*^) within LDA and LDA + DMFT. Within LDA, we find $({\delta }_{x},{\delta }_{y},{\delta }_{z})=(-1.12,-0.95,-\mathrm{5.22)}\,{\rm{eV}}$ and $({n}_{\sigma }^{x},{n}_{\sigma }^{y},{n}_{\sigma }^{z}=0.68,0.59,\mathrm{0.53)}$. LDA + DMFT severely renormalizes the center of gravity of each band to $({\delta }_{x},{\delta }_{y},{\delta }_{z})=(-3.76,-2.93,-\mathrm{6.65)}\,{\rm{eV}}$, as well as the orbital occupancies to $({n}_{\sigma }^{x},{n}_{\sigma }^{y},{n}_{\sigma }^{z}=0.69,0.78,\mathrm{0.56)}$, promoting enhanced orbital polarization. This fact, generic to multi-orbital systems (though we do not find total orbital polarization)^44^, is an interesting manifestation of correlation-induced orbital rearrangement, and controls structural changes across the Mott transition (see below).

**Figure 4:**
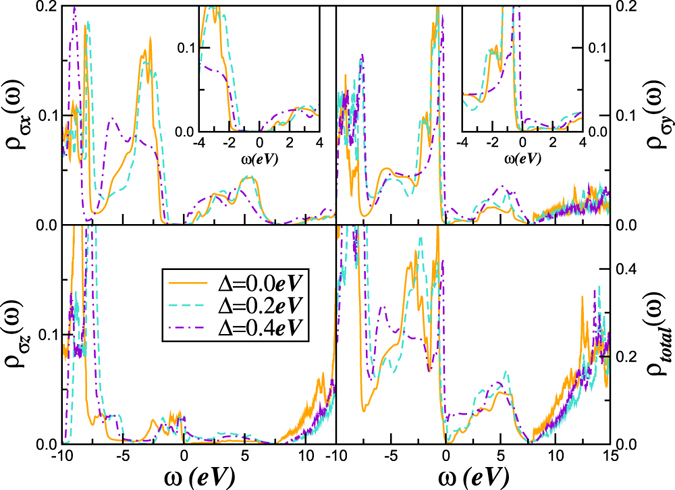
Orbital-selective insulator-to-metal transition in pressurized O_2_. In our theory, we vary the trial orbital-splitting Δ within LDA + DMFT to simulate structural changes upon pressure. In our results for the orbital-selective metallic phase the *p*_*y*_ (and hence, total) DOS shows a clear pseudogap around *E*_*F*_, corresponding to an orbital-selective, non-Fermi liquid metallic phase. Inset at the top panels show the evolution of the electronic states close to the Fermi energy.

**Figure 5:**
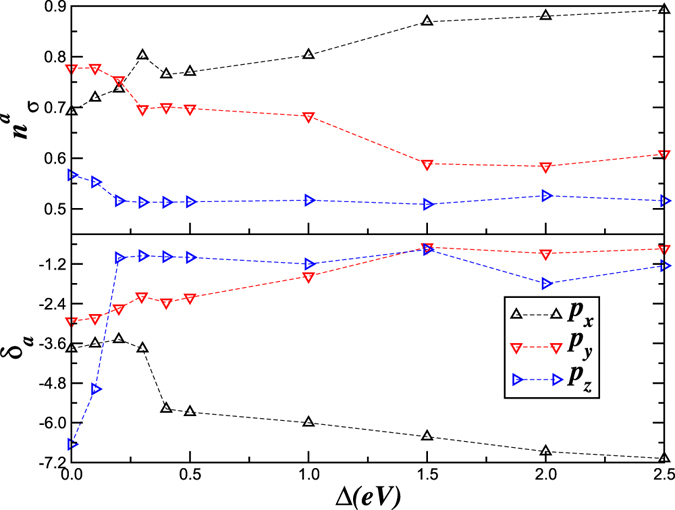
Effect of the orbital “Zeeman” field upon pressure. (Top) LDA + DMFT results for the orbital occupations ${n}_{\sigma }^{a}$ and (bottom) the renormalized orbital splittings *δ*_*a*_. Notice that ${n}_{\sigma }^{y}$ jumps at the insulator-metal transition, a behavior characteristic of orbital-selective Mott transition.

We now turn to the insulator-metal transition in solid O_2_ at high *P*, and adopt the following strategy to derive this transition. Instead of reverting back to the LDA to use a different LDA density-of-states corresponding to the metallic *ζ*-phase, we search for an instability of the insulating, *ε* phase to the paramagnetic-metal by varying *δ*_*a*_, found for the Mott insulator above. To proceed, consider the orbital-dependent on-site energy term, ${H}_{{\rm{\Delta }}}={\sum }_{i,a,\sigma }\,{{\rm{\Delta }}}_{a}{n}_{i\sigma }^{a}$ in our Hamiltonian. We now let the trial Δ vary in small steps, keeping Δ_*x*_ = −Δ, Δ_*y*_ = Δ, to simulate the structural (and hence, electronic) changes upon pressure. Here, Δ_*a*_ acts like an external field in the orbital sector (orbital fields), sensitively controlling the occupations of each orbital in much the same way as the magnetization of a paramagnet as function of an external magnetic, Zeeman field. However, in order to draw a qualitative interpretation of *H*_Δ_ in our total model Hamiltonian $\bar{H}=H+{H}_{{\rm{\Delta }}}$ and its relation to pressure-induced electronic reconstruction in solid O_2_, we recall that the pressure derivative of crystal-field splitting is given by $d{\rm{\Delta }}/dP=\xi {\rm{\Delta }}/K$, where *K* is the bulk modulus and *ξ* is a constant value^45^. This in turn suggests that in the pressure range of interest in this work solid O_2_ could be in a linear regime as observed in other materials under external pressure conditions^46, 47^. As for V_2_O_3_^39^, YTiO_3_^43^ and, more recently, for FeS^48^ we search for the second self-consistent LDA + DMFT solution by solving the multi-orbital DMFT equations for each trial value of Δ keeping *U*, *U*′ fixed. As seen in Fig. 4, small variations of Δ drive appreciable spectral weight transfer, producing drastic orbital-selective renormalizations of the one-particle spectral functions: the *p*_*x*_ and *p*_*y*_ are most severely affected. At a critical Δ_*c*_ = 0.3 eV, the *p*_*x*_ density-of-states remains Mott insulating, while the *p*_*y*_ band undergoes an insulator to bad-metal (weakly first-order, with no coherent Kondo peak at *E*_*F*_) transition. Thus, our results imply that the paramagnetic, metallic phase of *ζ*-oxygen is an orbital-selective incoherent metal without Landau quasiparticles, characterized by a pseudogap at *E*_*F*_ in the *p*_*y*_, and hence, in the total spectral function, at *E*_*F*_. Our simulations (not shown) indicate that Kondo-like resonance found below *E*_*F*_ for Δ = 0.4 eV will cross the Fermi level at extremely high pressures, driving solid O_2_ into (quasi)coherent Fermi liquid-like metallic state at even higher (experimentally uninvestigated) pressures. The underlying theoretical reason for this is as follows: At Δ_*c*_, strong scattering between the effectively (Mott) localized and itinerant components of the matrix DMFT propagators produces an incoherent metal because strong interband scattering indeed operates in a sizably orbitally polarized metallic system. However, at very high pressure (large Δ > Δ_*c*_), the *p*_*x*_ band becomes almost fully polarized (Fig. 5, upper panel) and the system evolves into a low-*T* correlated Fermi liquid metal^43^, which we predict to be the post-*ζ* phase. This is consistent with the fact that strong crystal-field splitting supresses local orbital fluctuations and cuts off the strong scattering channel. In turn, this controls the orbital-selective phase boundary of correlated multi-orbital systems^49^. From our results, the orbital-selective Mott phase is thereby suppressed at high pressure, leading to continuous evolution of the incoherent, bad-metal to a correlated Fermi liquid like metal.

Our results in Fig. 4 suggests that the charge carriers in solid O_2_ near the Mott insulator-to-metal transition have a dual nature, where effectively Mott localized *p*_*y*_ states co-exist with itinerant *p*_*z*_ electronic states at finite Δ. In this two-fluid scenario selective Mott localization of *p*_*y*_ states implies that this orbital now act like an intrinsic source of electronic disorder in the system. With Δ = 0.2 eV, for example, this suggests that an intrinsic disorder potential, arising from orbital-selective physics exists near the Mott transition. Such behavior results from strong scattering between effectively (Mott) localized and itinerant components of the full DMFT matrix propagators. This behavior is intimately linked to orbital-selective Mott-like physics within DMFT^50^.

In Fig. 5 we show the evolution of the orbital occupations ${n}_{\sigma }^{a}$ (top) and the renormalized orbital splittings *δ*_*a*_ across the insulator-metal transition. The features are well understood as follows. In a multiband situation, Δ acts like an external “Zeeman” field^39, 43^ in the orbital sector. The insulator-metal transition is characterized by a sudden jump in the renormalized *δ*_*z*_, and in the *p*_*x*_ and *p*_*y*_ populations as a consequence, suggesting that anisotropic structural (and volume) changes will accompany the orbital-selective Mott transtion. Here, we propose that these changes in *n* ^*a*^ control anisotropic changes in lattice parameters (**a**–**c**) across the insulator-metal transition: indeed, the changes in **a**–**c** are expressible in terms of *n* ^*a*^ as ${\gamma }_{a}={\rm{\Delta }}{l}^{a}/{l}^{a}=(\frac{g}{M{v}_{sa}^{2}})\,{\rm{\Delta }}{n}^{a}$, where *g* is the electron-phonon coupling constant, *M* the ion mass, and *v*_*sa*_ is the velocity of sound along *a*($\equiv$ *x*, *y*, *z*). Changes in *γ*_*a*_ across the insulator-to-metal transition thus follow those in the *n* ^*a*^. Though values of *v*_*sa*_ and *g* in the *ε* phase are unknown, we deduce that the lattice parameter **a** increases, while **b**,**c** descrease across the orbital-selective Mott transition as in Fig. 5 upper panel. The correct trend vis-a-vis experiment^10^ provides further support for our Mottness scenario in solid O_2_.

### Normal state resistivity

**Figure 6:**
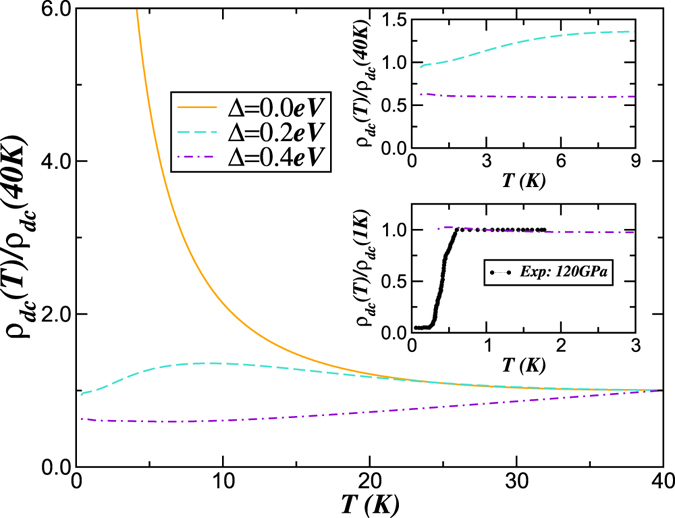
Electrical resistivity of solid O_2_ at high pressures. Main panel: Normal state resistivity versus temperature (normalized to *ρ*(40 *K*)), showing the metal-insulator transition with increasing the orbital “Zeeman” field Δ. Inset shows resistivity at very low temperatures: Observed features at low-*T* are well reproduced by strong Coulomb correlations *U*, *U*′ and Δ = 0.4 eV. Notice that results with small Δ value deviates from observation of constant *ρ*(*T*) at 120 GPa in experiment^9^. It is possible that a detailed experimental study of *ρ*_*dc*_(*T*) in the pressure range closer to the insulator-to-metal transition may reveal the trend we find, and this would constitute more concrete support for our modelling.

To illustrate the importance of correlation-induced changes in the orbital “Zemman” field Δ under high pressure in our theory, we now discuss our results for the normal state resistivity computed within the Kubo formalism^51^. In our theory, the observed features in *ρ*_*dc*_(*T*) originate from changes in the correlated spectral functions with Δ. Showing how this provides a compelling description of the admittedly limited available data is our focus in what follows. In Fig. 6, we show the *ρ*_*dc*_(*T*) for three values of Δ in solid O_2_, computed using the LDA + DMFT orbital resolved spectral functions (with *U* = 11.3 eV, *U*′ = *U* − 2*J*_*H*_, and *J*_*H*_ = 0.45 eV). Various interesting features immediately stand out. First, *ρ*_*dc*_(*T* → 0) in the *ε* phase (Δ = 0) shows semiconducting behavior, in accord with the insulating classification at lower pressures, when Δ < Δ_*c*_. Secondly, at all *T*, no Fermi liquid *T* ^2^-like contribution is detectable in the metallic phase with Δ = 0.4 eV: instead, *ρ*_*dc*_(*T*) is approximately constant up to 10 K. For intermediate pressure (but on the metallic side), *ρ*_*dc*_(*T*) crosses over from semiconductor-like (at high *T*) to bad-metallic (at low *T*) behavior. Remarkably, the detailed *T*-dependence closely resembles that seen in experiment^9^, in the normal state up to 2 K (see inset of Fig. 6). Since the system is proximate to a Mott transition, the *T*-dependence of *ρ*_*dc*_(*T*) for both values of Δ = 0.2, 0.4 eV is characteristic for carriers scattering off dynamically fluctuating and coupled, short-range spin and charge correlations. On general grounds, we expect this effect to be relevant near a correlation-driven Mott transition. Since an external magnetic field will generically quench spin fluctuations, we predict that destroying the *ε* Mott insulating state by a magnetic field^52^ might reveal this behavior. We emphasise that resistivity measurements as a function of pressure over extended *T* scales is a smoking gun for our proposal, as would be the study of the *T*-dependence of the *dc* Hall constant. These can distinguish a band-versus-Mott scenario for the pressure-induced insulator-metal transition: in the band-insulator-to-metal transition, there is no reason why, e.g., *ρ*_*dc*_(*T*) should show the above form, since neither orbital selectivity nor local antiferromagnetic spin fluctuations are operative there. More detailed transport work to corroborate our prediction are thus called for in future.

### Superconducting state in a selective pseudogapped metal

Insofar as superconductivity arises at the boundary of the Mott transition, our analysis provides tantalizing insight into sources of the pairing glue. Since the incoherent metal has a finite residual entropy ($S\propto ln\,2$ per site) from the Mott localized *p*_*x*_ sector, this electronic system is inherently unstable to soft two-particle instabilities^53^. In solid O_2_ at high pressure, lack of conditions supporting magnetic and charge-density instabilities in the correlated electronic structure near the insulator-metal transition opens the door to superconductivity as the only two-particle instability that can quench this finite normal state entropy. In fact, the situation is quite similar to that considered by Capone *et al*.^54^ in the fulleride context, where the pseudogapped, bad metal arose from an unstable (intermediate coupling) fixed point in the impurity problem, corresponding to the Kondo unscreened phase: in our case, precisely the same effect results from selective localization of the *p*_*x*_ band at the orbital-selective Mott phase. In fact, in the orbital-selective metal, the low-energy physics is selfconsistently controlled by strong scattering between quasi-itinerant *p*_*y*,*z*_ and Mott localized *p*_*x*_ orbital states, implying low-energy singularities in one- and two-particle propagators^55^. This suggests soft, multi-orbital electronic modes at low energy, which can potentially act as a pair glue. In a way similar to the fulleride case, we then expect that multiband spin-singlet *s*-wave superconductivity (notice that ${J}_{H}\ll U$, favoring *S* = 0), driven by such soft inter-orbital electronic fluctuations in this unstable phase, will cut off the incoherent metal found above, and that the superconducting transition temperature *T*_*c*_ will rise to values larger than those obtained for the weakly correlated case^54^. Interestingly, the variation of *T*_*c*_ with decreasing *U*/*W* (increasing pressure) found by Capone *et al*. does bear uncanny resemblance (Fig. 4 of ref. 54) to the *T*_*c*_(*P*) observed in solid O_2_ under high pressure. This is suggestive, but out of scope of the present work. We leave details for the future.

Our findings put constraints on mechanisms of superconductivity in solid O_2_. In view of the complexity of the problem (as discussed below), we only restrict ourselves to a qualitative discussion. Given selective incoherent metallic state within DMFT in our case, residual, inter-site and inter-orbital (in multiband systems) two-particle interactions can generate ordered states directly from the bad metal. Following the philosophy used earlier^53^ for the iron-pnictides superconducting systems we restrict ourselves to the *y*, *z* orbital sector. In this situation the interaction in the Cooper channel reads ${H}_{pair}=\frac{1}{2}{\sum }_{a,b,k,k^{\prime} }\,{V}_{ab}(k,k^{\prime} ){c}_{a,k,\uparrow }^{\dagger }{c}_{b,-k,\downarrow }^{\dagger }{c}_{b,-k^{\prime} ,\downarrow }{c}_{a,k^{\prime} ,\uparrow }$, where *a*, *b* = *y*, *z* and the scattering vertex is ${V}_{ab}(k,k^{\prime} ,\omega )={g}^{2}{\chi }_{ab}(k-k^{\prime} ,\omega )$ with ${\chi }_{ab}(k-k^{\prime} ,\omega )$ being the inter-orbital susceptibility. Decoupling *H*_*pair*_ in the particle-particle channel using Gorkov’s formalism^56^ gives ${H}_{pair}^{MF}={\sum }_{abk}\,[{{\rm{\Delta }}}_{ab}(k)({c}_{ka\uparrow }^{\dagger }{c}_{-kb\downarrow }^{\dagger }+h.c)]$, where ${{\rm{\Delta }}}_{ab}(k)=\frac{1}{2}{V}_{ab}\langle {c}_{-kb\downarrow }{c}_{ka\uparrow }\rangle$. This represents an inter-orbital pairing instability of the normal bad metal state near the Mott transition in pressurized O_2_. A plausible assuption to be made here is that the superconducting gap function has no nodes and therefore it can be taken to be momentum independent, i.e., ${{\rm{\Delta }}}_{ab}(k)\equiv {{\rm{\Delta }}}_{SC}$, for the multiband *s*-wave case of solid O_2_. However, as shown below this assumption has profound effects in the multiband excitation spectrum withing the *s*-wave superconducting phase.

**Figure 7:**
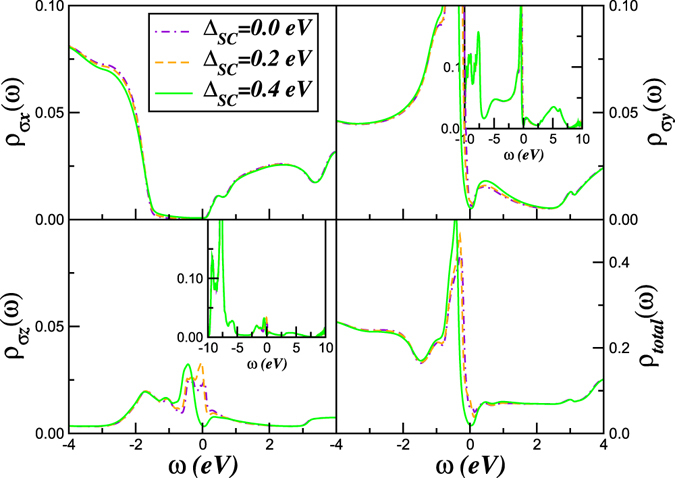
Orbital resolved and total LDA + DMFT density-of-states (DOS) in the normal (Δ_*SC*_ = 0) and supercoducting state $({{\rm{\Delta }}}_{SC}\ne 0)$ of solid O_2_. Here, the normal state LDA + DMFT solution for Δ = 0.4 eV is used as starting point towards understanging the selective modification of the excitation spectrum within the superconducting state. Notice the evolution of the superconducting gap at low energies in the electronic DOS of the *p*_*y*_ and *p*_*z*_ orbtials.

Aiming to shine light on the changes in the excitation spectrum of solid O_2_ across the superconducting phase transition we have extented our normal state electronic structure calculation to treat *H*_*pair*_ above within LDA + DMFT formalism for the superconducting state^53^. In fact, using our assumption for the *s*-wave superconducting gap function Δ_*SC*_ the LDA + DMFT equations are readily extendable to the superconducting regime. As in ref. 53 the one-particle Green’s function (*G*_*ab*_) have normal and anomalous components yielding remormalized *G*_*aa*_ propagators, which are solved by extending the normal state LDA + DMFT solution to include an explicit pair-field term. Including the pair-field, the DMFT propagators are writen as ref. 53 ${G}_{aa}(k,\omega )={[\omega -{\varepsilon }_{ka}-{{\rm{\Sigma }}}_{a}(\omega )-\frac{{{\rm{\Delta }}}_{ab}^{2}(k)}{\omega +{\varepsilon }_{kb}+{\Sigma }_{b}^{\ast }(\omega )}]}^{-1}$, where the $\ast$ denotes complex conjugation. However, since these equations couple all *p*-orbitals of oxigen, the opening up of a superconducting gap in a particular *p*-band might induce secondary gaps in the remaining *p* orbitals, in a way reminiscent of the inter-band proximity effect induced by *U*′ and *J*_*H*_ in multiband supeconductors. With this caveats in mind, we now describe our results within the superconducting state of solid O_2_. Using the normal state LDA + DMFT solution for Δ = 0.4 eV (a value which provides good agreement with resistivity data in the normal state, Fig. 6), the one-electron spectral functions can be read off and used for comparison with observables in the normal and superconducting states. Figure 7, therefore, show the changes induced by superconductivity in the orbital-resolved and total spectral functions of O_2_ across the normal-to-superconducting state. As seen the *p*_*z*_ channel is the most affect by the pairing mechanism. Clear appearence of a superconducting gap and sharp singularities at low-energies is seen across the superconduciting instability. However, normal state incoherence in the equation for *p*_*y*_ propagator [*G*_*yy*_(*k*, *ω*)] prevents opening up of a clean superconducting gap in the electronic spectrum. Remarkable as well is the fact that the insulating *p*_*x*_ channel is not affected at low energies by the superconducting instability, and this is strictly related to the fact that in this electronic channel the excitation spectrum is already gapped in the normal state. Moreover, weak spectral weight transfer from low- to high-energies occurs in all *p* bands as seen in Fig. 7. This suggests an orbital selective coupling of the carrier propagators to multi-orbital, overdamped charge- and spin-excitations which could be tested in future resonant inelastic x-ray scattering studies^57^. Future tunneling spectroscopy (*dI*/*dV*) measurements are also called for to corroborate our predition for the changes in the total one-particle spectral function (Fig. 7 right-lower panel) across the superconducting phase transition in solid O_2_.

## Conclusion

In conclusion, we have theoretically studied the insulator-metal transition in highly pressurized solid O_2_ using first-principles local-density-approximation plus dynamical-mean-field calculations. In analogy with multi-orbital *d*- and *f*-band systems, we find an orbital-selective Mott transition and an incoherent metallic normal state, arising from the Mott insulator via a weakly first-order transition as a function of pressure. Implications of our picture for the superconducting state are discussed: we propose that soft, multi-orbital electronic fluctuations involving dualistic states, i.e., the quasi-itinerant (*p*_*y*_, *p*_*z*_) and Mott localized (*p*_*x*_) states arising at this orbital-selective Mott transition act as the pairing glue for the superconducting state found at low *T* in solid O_2_. Our work underlines the importance of local dynamical correlations in this molecular-solid system, and holds promise for understanding similar physics in other solidified gases.

## Methods

To unveil the electronic reconstruction at the border of the Mott metal-insulator transition in solid Oxygen, we employ an implementation of the local density plus dynamical mean-field (LDA + DMFT) scheme^31^, which correctly takes disorder, temperature and pressure effects into account in numerous strongly correlated multiband systems^39^. Within our scheme, the LDA band structure calculations were performed for the experimental crystal (*C*2/*m*) structure of solid *O*_2_ using the non-fully relativistic version of the PY-LMTO code^34, 35^. To incorporate the effects of dynamical electronic correlations in solid O_2_, we use the multi-orbital iterated-perturbation-theory (MO-IPT) as an impurity solver of the many-particle problem in DMFT^38, 39, 58^. This method has recently been benchmarked by comparison with merically exact continuous time quantum Monte Carlo method (CTQMC) as an impurity solver in DMFT^58^. These authors find very good accord between MO-IPT and CTQMC for both one-band and multi-band Hubbard models when realistic, non-negligible crystal field splittings are included. Thus, with sizable effects of the real crystal field splitting in solid O_2_, our IPT solver is sufficiently accurate for quantifying the effects of sizable electronic correlations in solid O_2_. LDA + DMFT is thus to be viewed as a combined density functional plus dynamical mean-field (DMFT) scheme^31^, and adequately describes the effect of local dynamical interactions in the limit of large lattice dimensions (DMFT)^59^. Thereby, practical calculations become feasible even for complex multiband systems like solid O_2_. The basic idea in a DMFT solution involves replacing the lattice model by a self-consistently embedded MO-Anderson impurity model, and the self-consistency condition requiring the local impurity Green’s function to be equal to the local Green’s function for the lattice. The full set of equations for the MO case can be found in refs 38, 39, 58, so we do not repeat the equations here. It worth mentioning, however, that the IPT is an interpolative *ansatz* that connects the two exactly soluble limits of the one-band Hubbard model^60^, namely, the uncorrelated (*U* = 0) and the atomic (*ε*_**k**_ = 0) limits. At intermediate *U*, it ensures the correct low-energy behavior of the self-energies and spectral functions by strict observance of the Friedel-Luttinger sum rule in the impurity solver (we have used MO-IPT). It thus accounts for the correct low- and high-energy behavior of the one-particle spectra by construction. It ensures the Mott-Hubbard metal-insulator transition from a correlated FL metal to a Mott-Hubbard insulator occurs as a function of the Coulomb interaction *U*. Compared to numerically expensive QMC solvers, IPT-based schemes are known to be computationally very efficient, and readily yield real frequency data at zero and finite temperatures without the need to perform numerical analytic continuations, which are known to be a very delicate matter in QMC schemes. Finally, we have carried out the computation of electrical transport within the Kubo formalism^51^.
